# *TAS2R38* haplotypes, COVID-19 infection, and symptomatology: a cross-sectional analysis of data from the Canadian Longitudinal Study on Aging

**DOI:** 10.1038/s41598-024-55428-4

**Published:** 2024-02-26

**Authors:** Tongzhu Meng, Daiva E. Nielsen

**Affiliations:** School of Human Nutrition, McGill University, 21,111 Lakeshore Rd., Room MS2-035, Saint-Anne-de-Bellevue, QC H9X 3V9 Canada

**Keywords:** Haplotypes, Viral infection

## Abstract

The *TAS2R38* gene is well known for its function in bitter taste sensitivity, but evidence also suggests a role in innate immunity. *TAS2R38* may be relevant in coronavirus disease 2019 (COVID-19), but research findings are inconsistent. The objective of this study was to explore whether common *TAS2R38* haplotypes are associated with COVID-19 infection and symptomatology in the Canadian Longitudinal Study on Aging (CLSA). Data from the CLSA COVID-19 Questionnaire and Seroprevalence sub-studies were utilized with CLSA genetic data for common *TAS2R38* haplotypes related to bitter taste sensitivity. Haplotypes were categorized into three diplotype groups: [P]AV homozygotes, [P]AV/[A]VI heterozygotes, and [A]VI homozygotes. No significant differences were observed between diplotypes and COVID-19 infection frequency. Among self-reported COVID-19 cases (n = 76), and in uncorrected exploratory analyses, heterozygotes were less likely to report experiencing sinus pain compared to [P]AV homozygotes. Among seroprevalence-confirmed cases (n = 177), [A]VI homozygotes were less likely to report experiencing a sore/scratchy throat compared to [P]AV homozygotes. However, both observations were non-significant upon correction for multiple testing. In this study, *TAS2R38* haplotypes were not significantly associated with COVID-19 infection or symptomatology. Nevertheless, in light of some exploratory patterns and conflicting evidence, additional research is warranted to evaluate links between *TAS2R38* and innate immunity.

## Introduction

The ability of humans to sense bitter taste is thought to have evolved as a warning signal to avoid consuming toxins^1^. The bitter taste sensations are facilitated by bitter taste receptors that detect a range of bitter compounds with different chemical structures^2^. Bitter taste receptors in the oral cavity are encoded by bitter taste receptor genes in the human genome and variation present in these functional genes results in individual variability to sense bitter taste. The locus *TAS2R38*, which encodes taste receptor type 2 (T2R) member 38, is the most widely studied bitter taste receptor gene. Three single nucleotide polymorphisms (rs713598, rs1726866 and rs10246939) at positions encoding amino acids 49, 262 and 296 give rise to two common haplotypes, PAV and AVI, which are known to alter sensitivity to the bitter taste of phenylthiocarbamide (PTC) or the structurally related compound 6-n-propylthiouracil (PROP)^3,4^. The PAV homozygous and PAV/AVI heterozygous diplotypes (combinations of two haplotypes) generally relate to more sensitivity to PTC and PROP compared to the AVI homozygous diplotype^3,5^. However, while these common *TAS2R38* haplotypes correlate well with PTC/PROP threshold phenotype, other factors are involved in the perception of PROP bitterness intensity (e.g. differences in oral anatomy)^6,7^.

While a role of *TAS2R38* in PTC/PROP sensitivity is well established, the expressions of T2R38 have also been found in extra-oral tissues such as the airway, and emerging evidence suggests a potential role of T2R38 in upper respiratory (comprised of the sinonasal cavity) innate immunity^8^. T2R38 activation by its agonist or microbe-derived quorum-sensing molecules leads to the release of nitric oxide (NO), which helps to increase ciliary beating frequency and has direct antibacterial effects^9^. A previous study showed that sinonasal epithelial cells cultured from PAV homozygotes had higher NO release along with faster ciliary beating frequency and mucociliary clearance compared to cells cultured from AVI homozygotes^9^. Furthermore, the role of *TAS2R38* in innate immunity in response to pathogens of the respiratory tract may be supported by recently reported associations between *TAS2R38* haplotypes (or T2R38 phenotypes) and coronavirus disease 2019 (COVID-19) infection and symptoms, although evidence is mixed^10–13^.

Ecological evidence using pooled data from several countries previously linked a higher presence of the *TAS2R38* PAV haplotype than AVI with lower COVID-19 mortality, suggesting that sensitivity to PTC/PROP may confer protective effects^11^. Moreover, in a prospective cohort study, Barham et al. assessed T2R38 phenotype using taste strips for PTC, thiourea, and sodium benzoate (as well as a chemical free control strip) and evaluated associations between bitter taster phenotypes and COVID-19 outcomes from medical records^12^. Individuals exhibiting the “non-taster” T2R38 phenotype were more likely to be infected with COVID-19 than “taster” phenotypes and experience severe symptomatology. However, such associations were not observed in a smaller cross-sectional study conducted by Risso et al., who reported that COVID-19 outcomes did not differ according to PAV or AVI *TAS2R38* haplotypes or PROP “taster” and “non-taster” phenotypes^13^. In addition, to our knowledge, genome-wide association studies (GWAS) of COVID-19 infection, severity, and symptomatology have not reported *TAS2R38* as a locus of interest^14–16^. Nevertheless, given the mixed evidence and knowledge of the gene’s plausible role in innate immunity, further investigation in the context of COVID-19 is warranted. Therefore, the objective of the present study was to explore whether common *TAS2R38* haplotypes are associated with COVID-19 infection and symptomatology in the Canadian Longitudinal Study on Aging (CLSA).

## Results

Out of 28,565 respondents in the CLSA COVID-19 Questionnaire Study, a total of 13,825 CLSA respondents were included in the present investigation. Participants were excluded from analyses (n = 14,740) due to missing responses to the Exit Questionnaire survey, non-participation in the CLSA Comprehensive Cohort, or missing required genetic data (Supplementary Fig. S1). Within the included respondents, n = 2967 were classified as [P]AV homozygotes based on their diplotypes while n = 6806 and n = 4052 were classified as heterozygotes and [A]VI homozygotes, respectively (Table 1).

**Table 1:** Characteristics of respondents in COVID-19 Questionnaire Study and COVID-19 Seroprevalence Study.

	COVID-19 Questionnaire Study			COVID-19 Seroprevalence Study		
	[P]AV homozygotes (n = 2967)	Heterozygotes (n = 6806)	[A]VI homozygotes (n = 4052)	[P]AV homozygotes (n = 1893)	Heterozygotes (n = 4321)	[A]VI homozygotes (n = 2577)
Sex						
Males	1431 (48.2%)	3272 (48.1%)	1963 (48.5%)	922 (48.7%)	2166 (50.1%)	1289 (50.0%)
Females	1536 (51.8%)	3534 (51.9%)	2089 (51.5%)	971 (51.3%)	2155 (49.9%)	1288 (50.0%)
Age	68.8 ± 9.4	68.9 ± 9.4	68.8 ± 9.4	69.4 ± 9.3	69.0 ± 9.3	69.0 ± 9.3
BMI (kg/m^2^)	28.0 ± 5.2	27.9 ± 5.2	27.9 ± 5.2	26.8 ± 1.2	26.8 ± 1.2	27.0 ± 1.2
Self-reported Ethnicity						
Caucasians	2864 (96.5%)	6613 (97.2%)	3957 (97.7%)	1783 (94.8%)	4135 (96.3%)	2481 (96.6%)
Non-Caucasians	103 (3.5%)	193 (2.8%)	95 (2.3%)	98 (5.2%)	159 (3.7%)	87 (3.4%)
Current smoking status						
Current smoker	165 (5.6%)	416 (6.2%)	229 (5.7%)	81 (4.3%)	191 (4.5%)	119 (4.7%)
Non-smoker	2772 (94.4%)	6313 (93.8%)	3780 (94.3%)	1785 (95.7%)	4082 (95.5%)	2439 (95.3%)
Highest level of education						
Less than secondary school graduation	124 (4.2%)	270 (4.0%)	166 (4.1%)	71 (3.8%)	163 (3.8%)	111 (4.4%)
Secondary school graduation, no post-secondary graduation	458 (15.5%)	1096 (16.1%)	596 (14.7%)	279 (15.0%)	622 (14.6%)	379 (14.9%)
Post-secondary degree/diploma	2380 (80.3%)	5433 (79.9%)	3285 (81.2%)	1516 (81.2%)	3473 (81.6%)	2062 (80.8%)

Bracketed letters indicate the inferred variant in the current study. Values presented are n (%) except for age and body mass index (BMI), which are presented as mean ± standard deviation. Responses to sex, age and current smoking status used in the COVID-19 Questionnaire Study were assessed in the COVID-19 Baseline Questionnaire. Self-reported ethnicity, highest level of education and BMI used in in the COVID-19 Questionnaire Study were assessed in the CLSA Baseline Comprehensive Cohort assessment. Missing responses for COVID-19 Questionnaire Study included missing current smoking status (n = 150), did not specify education status (n = 17) and missing BMI (n = 24). Missing responses for COVID-19 Seroprevalence Study included missing current smoking status (n = 94), did not specify education status (n = 115), missing BMI (n = 319) and missing self-reported ethnicity (n = 48).

Across the three diplotypes, the average age (mean) was 68.8 years old with baseline BMI of 28.0 kg/m^2^. The sample consisted of approximately similar proportion of sex groups across the three diplotypes and the majority of the sample was highly educated, non-smoking, and Caucasian. Included respondents were of a similar age and education level compared to the excluded respondents, but excluded respondents were more likely to be female and non-Caucasian, have a higher BMI and be current smokers compared to the included respondents. The proportion of respondents with common COVID-19 comorbidities varied between participants included and excluded from the investigation (Supplementary Table S2). Diabetes and hypertension were more prevalent among the included participants, while asthma was less prevalent.

Out of all included respondents, n = 76 respondents self-reported having a confirmed COVID-19 infection (0.5%). Among these respondents considered as COVID-19 cases, asthma was significantly more prevalent among [P]AV homozygotes compared to heterozygotes and [A]VI homozygotes, while the prevalence of other common COVID-19 comorbidities was not significantly different across diplotypes (Table 2). While COVID-19 cases were highest among heterozygotes, no significant difference was observed between diplotypes and COVID-19 infection frequency using Fisher’s exact test (n [%]: [P]AV homozygotes 20 [26.3%], heterozygotes 34 [44.7%], [A]VI homozygotes 22 [29.0%], *P* = 0.56). The same non-significant result was observed with multivariable logistic regression (odds ratio [95% confidence interval]: [P]AV homozygotes 1.0 (reference), heterozygotes 0.74 [0.42, 1.28], [A]VI homozygotes 0.81 [0.44, 1.49], *P* = 0.56).

**Table 2:** Common comorbidities of respondents with COVID-19 infection.

	COVID-19 Questionnaire Study					COVID-19 Seroprevalence Study				
	[P]AV homozygotes (n = 20)	Heterozygotes (n = 34)	[A]VI homozygotes (n = 22)	*p*-value	FDR adjusted *p*-value	[P]AV homozygotes (n = 37)	Heterozygotes (n = 76)	[A]VI homozygotes (n = 64)	*p*-value	FDR adjusted *p*-value
Asthma	10^a^	7^b^	2^b^	0.007	0.049	4	8	4	0.62	0.69
Chronic Lung Diseases	2	1	1	0.69	0.91	2	2	3	0.69	0.69
Diabetes	0	4	4	0.19	0.67	7	7	5	0.19	0.44
Hypertension	3	9	7	0.44	0.91	15	16	17	0.09	0.32
Heart diseases	2	4	1	0.78	0.91	5	6	3	0.29	0.51
Cancer	1	2	1	1.00	1.00	8	11	9	0.55	0.69
Immune suppressed	1	4	1	0.65	0.91	3^a^	0^b^	0^b^	0.009	0.06

Bracketed letters indicate the inferred variant in the current study. Values presented are number of respondents from unadjusted Pearson Chi-square tests or Fisher’s Exact tests where appropriate. Immune suppressed in the COVID-19 Questionnaire Study included having autoimmune disease/HIV, received an organ, bone marrow or stem cell transplant. Different letter superscripts indicate statistically different pairwise comparison values.

Among participants who self-reported a confirmed COVID-19 infection, a lower proportion of heterozygotes reported experiencing sinus pain compared to [P]AV homozygotes, but this observation was non-significant upon false discovery rate (FDR) correction for multiple testing (Table 3). No significant differences were observed for other symptomatology. The results from the unadjusted models using Fisher’s exact test and adjusted logistic regression models were in accordance with each other for all symptomatology outcome variables (Table 4). The multivariable regression models indicated that heterozygotes were 86% less likely to report sinus pain compared to [P]AV homozygotes, but the finding was not statistically significant upon FDR correction.

**Table 3:** Comparisons of experience of COVID-19 symptomatology by *TAS2R38* diplotypes among respondents with COVID-19 infection.

	COVID-19 Questionnaire Study					COVID-19 Seroprevalence Study				
	[P]AV homozygotes (n = 20)	Heterozygotes (n = 34)	[A]VI homozygotes (n = 22)	*p*-value	FDR adjusted *p*-value	[P]AV homozygotes (n = 37)	Heterozygotes (n = 76)	[A]VI homozygotes (n = 64)	*p*-value	FDR adjusted *p*-value
Fever	7	14	9	0.92	0.93	2	4	3	1.0	1.0
Cough	–	–	–	–	–	7	10	10	0.77	0.90
Dry cough	12	22	12	0.75	0.93	–	–	–	–	–
Wet cough	6	8	9	0.42	0.93	–	–	–	–	–
Shortness of breath	9	19	8	0.29	0.93	4	9	4	0.50	0.90
Decreased sense of smell	7	16	9	0.62	0.93	1	3	1	0.68	0.90
Fatigue	17	26	20	0.65	0.93	–	–	–	–	–
Sore/scratchy throat	12	19	12	0.93	0.93	7^a^	11^a^	2^b^	0.03	0.21
Sore muscles or muscle pain	12	17	11	0.80	0.93	6	9	10	0.77	0.90
Headache	14	19	11	0.42	0.93	9	14	16	0.64	0.90
Running nose	10	16	12	0.86	0.93	–	–	–	–	–
Sinus pain	9^a^	4^b^	6^ab^	0.03	0.36	–	–	–	–	–
Feeling unwell	9	16	10	0.92	0.93	–	–	–	–	–

Bracketed letters indicate the inferred variant in the current study. Values presented are number of respondents from unadjusted Pearson Chi-square tests or Fisher’s Exact tests where appropriate. Different letter superscripts indicate statistically different pairwise comparison values. Responses to the experience of COVID-19 symptomatology used in the COVID-19 Questionnaire Study were assessed in the COVID-19 Exit Questionnaire. Missing responses for COVID-19 Questionnaire Study included n = 3 did not respond to fever; n = 1 did not respond to wet cough; n = 1 did not respond to shortness of breath; n = 3 did not respond to decreased sense of smell, n = 2 did not respond to fatigue; n = 2 did not respond to sore muscles or muscle pain; n = 1 did not respond to headache; n = 2 did not respond to sinus pain and n = 2 did not respond to feeling unwell. Missing responses for COVID-19 Seroprevalence Study included n = 2 did not respond to fever; n = 3 did not respond to cough; n = 2 did not respond to shortness of breath; n = 2 did not respond to decreased sense of smell; n = 3 did not respond to sore/scratchy throat and n = 3 did not respond to headache.

**Table 4:** Odds ratios (95% confidence intervals) of experience of COVID-19 symptomatology by *TAS2R38* diplotypes among respondents with COVID-19 infection.

	COVID-19 Questionnaire Study					COVID-19 Seroprevalence Study				
	[P]AV homozygotes (n = 20)	Heterozygotes (n = 34)	[A]VI homozygotes (n = 22)	*p*-value	FDR adjusted *p*-value	[P]AV homozygotes (n = 37)	Heterozygotes (n = 76)	[A]VI homozygotes (n = 64)	*p*-value	FDR adjusted *p*-value
Fever	1.0 (Ref)	1.31 (0.38, 4.50)	1.35 (0.35, 5.30)	0.89	0.89	1.0 (Ref)	0.55 (0.07, 4.56)	0.63 (0.08, 5.26)	0.85	0.85
Cough	–	–	–	–	–	1.0 (Ref)	0.46 (0.15, 1.49)	0.53 (0.16, 1.72)	0.41	0.75
Dry cough	1.0 (Ref)	1.12 (0.33, 3.84)	0.66 (0.18, 2.50)	0.67	0.89	–	–	–	–	–
Wet cough	1.0 (Ref)	0.70 (0.19, 2.60)	1.79 (0.46, 7.01)	0.34	0.89	–	–	–	–	–
Shortness of breath	1.0 (Ref)	1.62 (0.49, 5.38)	0.63 (0.17, 2.39)	0.29	0.89	1.0 (Ref)	1.00 (0.27, 3.80)	0.51 (0.12, 2.29)	0.54	0.75
Decreased sense of smell	1.0 (Ref)	1.83 (0.50, 6.77)	1.90 (0.45, 7.96)	0.60	0.89	1.0 (Ref)	1.91 (0.17, 21.18)	0.65 (0.04, 11.61)	0.64	0.75
Fatigue	1.0 (Ref)	0.90 (0.17, 4.80)	1.80 (0.22, 15.07)	0.75	0.89	–	–	–	–	–
Sore/scratchy throat	1.0 (Ref)	0.69 (0.21, 2.35)	0.64 (0.17, 2.44)	0.78	0.89	1.0 (Ref)	0.59 (0.18, 1.96)	0.11 (0.02, 0.61)	0.04	0.28
Sore muscles or muscle pain	1.0 (Ref)	0.58 (0.16, 2.17)	0.36 (0.09, 1.53)	0.38	0.89	1.0 (Ref)	0.55 (0.17, 1.82)	0.85 (0.27, 2.72)	0.56	0.75
Headache	1.0 (Ref)	0.61 (0.17, 2.19)	0.50 (0.12, 2.00)	0.60	0.89	1.0 (Ref)	0.44 (0.15, 1.26)	0.73 (0.26, 2.04)	0.27	0.75
Running nose	1.0 (Ref)	0.79 (0.25, 2.53)	1.15 (0.32, 4.14)	0.80	0.89	–	–	–	–	–
Sinus pain	1.0 (Ref)	0.14 (0.03, 0.61)	0.43 (0.11, 1.74)	0.03	0.36	–	–	–	–	–
Feeling unwell	1.0 (Ref)	1.36 (0.40, 4.57)	0.98 (0.26, 3.69)	0.83	0.89	–	–	–	–	–

Bracketed letters indicate the inferred variant in the current study. Values are odds ratios (95% confidence interval) estimated from multivariable logistic model adjusted for age, sex and first five principal components of ancestry. Responses to the experience of COVID-19 symptomatology used in the COVID-19 Questionnaire Study were assessed in the COVID-19 Exit Questionnaire. Missing responses for COVID-19 Questionnaire Study included n = 3 did not respond to fever; n = 1 did not respond to wet cough; n = 1 did not respond to shortness of breath; n = 3 did not respond to decreased sense of smell, n = 2 did not respond to fatigue; n = 2 did not respond to sore muscles or muscle pain; n = 1 did not respond to headache; n = 2 did not respond to sinus pain and n = 2 did not respond to feeling unwell. Missing responses for COVID-19 Seroprevalence Study included n = 2 did not respond to fever; n = 3 did not respond to cough; n = 2 did not respond to shortness of breath; n = 2 did not respond to decreased sense of smell; n = 3 did not respond to sore/scratchy throat and n = 3 did not respond to headache.

Patterns of associations were further examined among the Seroprevalence Study sample. Out of 19,334 respondents in the CLSA COVID-19 Seroprevalence Study, n = 8791 were included in the present analyses. Participants were excluded (n = 10,543) due to missing required genetic data and SARS-CoV-2 infection-induced seroprevalence data (Supplementary Fig. S2). The most common reason for exclusion from analyses was lack of genetic data. Among the included respondents, n = 1893 were [P]AV homozygotes while n = 4321 and n = 2577 were heterozygotes and [A]VI homozygotes, respectively.

The included respondents in the COVID-19 Seroprevalence Study had a mean age of approximately 69.0 years with BMI 27.0 kg/m^2^ and similar proportion of sexes between diplotypes (Table 1). The majority were non-smoking Caucasians with a post-secondary degree of education. Comparing characteristics of participants included versus excluded from the present investigation, excluded participants were more likely to be female, have a higher BMI, be of non-Caucasian ethnicity, a current smoker, and of lower education status. Moreover, a greater proportion of excluded respondents had COVID-19 comorbidities including chronic lung diseases, diabetes, heart diseases, cancer, and diseases that suppress the immune system.

Among the included respondents, n = 177 were considered as having a confirmed COVID-19 infection based on their SARS-CoV-2 infection-induced seroprevalence result (2.0%). The common COVID-19 comorbidities did not differ significantly by diplotypes upon FDR correction (Table 2). None of the diplotypes was significantly associated with COVID-19 infection frequency using Fisher’s exact test (n [%]: [P]AV homozygotes 37 [20.9%], heterozygotes 76 [42.9%], [A]VI homozygotes 64 [36.2%], *P* = 0.11). This was also observed in the multivariable logistic regression model (odds ratio [95% confidence interval]: [P]AV homozygotes 1.0 (reference), heterozygotes 0.91 [0.61, 1.35], [A]VI homozygotes 1.33 [0.88, 2.01], *P* = 0.08). While a lower proportion of [A]VI homozygotes reported experiencing a sore/scratchy throat compared to [P]AV homozygotes and heterozygotes in uncorrected analyses, this finding was non-significant upon FDR correction (Table 3). The results from unadjusted models using Fisher’s exact test and odds ratio estimated from adjusted logistic regression models were in accordance with each other and the adjusted regression model indicated that [A]VI homozygotes were 89% less likely to report a sore/scratchy throat compared to [P]AV homozygotes, although the observation did not remain statistically significant upon FDR correction (Table 4). No other symptomatology outcome variables differed by diplotypes.

## Discussion

This study aimed to explore associations between common *TAS2R38* haplotypes and COVID-19 infection and symptomatology among middle-aged and older-aged adults in the CLSA. By analysing self-reported questionnaire data and objective SARS-CoV-2 infection-induced seroprevalence data, COVID-19 infection status was not found to be associated with common *TAS2R38* haplotypes. In uncorrected exploratory analyses, *TAS2R38* haplotypes were associated with reports of symptoms relevant to upper respiratory innate immunity, including sinus pain and sore/scratchy throat among COVID-19 cases. However, these observations were non-significant upon FDR correction and there were no observed associations for the other symptomatology, including some of the widely discussed symptoms of COVID-19 infection including loss of smell, fever, and cough^17^.

Our observations of a general lack of relationship between *TAS2R38* haplotypes and COVID-19 outcomes are supported by GWAS literature, which to our knowledge have not identified *TAS2R38* as a locus of interest for COVID-19 infection or severity^14,15^. Moreover, even GWAS of distinctive COVID-19 symptoms, such as loss of taste and smell, have not identified *TAS2R38* variants as significant loci^16^. Therefore, the common *TAS2R38* haplotypes studied in this present investigation appear not to be linked with COVID-19 susceptibility/severity. However, it is worth noting that prior to correcting for multiple statistical testing, the present study observed some associations between *TAS2R38* diplotypes and self-reported experience of certain COVID-19 symptoms related to the sinuses and throat. Given the limited number of COVID-19 cases identified in the present study along with the biological plausibility of a role of *TAS2R38* in innate immunity, these observations are worth reporting (with caution) to inform future research in this area. Yet it is important to consider that any potential associations between common *TAS2R38* haplotypes and viral infection symptomatology may reflect the gene’s involvement in upper respiratory innate immunity more generally as opposed to being uniquely linked to COVID-19. Thus, it is conceivable that individuals who carry the same *TAS2R38* diplotypes may exhibit common patterns in symptomatology arising from a broad range of viral infections, but further research is necessary to evaluate this.

A careful examination of prior research examining *TAS2R38* (or its phenotypic expression) and COVID-19 is needed to properly situate the results reported presently. Although ecological evidence has suggested a link between *TAS2R38* haplotypes and COVID-19 mortality^11^, the observation must be interpreted with caution due to the likelihood of residual confounding^18^. However, in a previous study conducted by Barham et al., T2R38 phenotypes were reported to be significantly associated with SARS-CoV-2 infection and severity^12^. Participants were n = 1935 patients and healthcare workers from a tertiary outpatient clinical practice and inpatient hospital in the United States who had exposure to SARS-CoV-2 (n = 266 were COVID-19 cases with a positive PCR test result). T2R38 taste receptor phenotype was categorized based on phenotype testing using taste strips that are associated with *TAS2R38* haplotypes^19^. Bitter “non-tasters” were found to be significantly more likely than bitter “taster” phenotypes to be infected with COVID-19, to be hospitalized after the infection, and to be symptomatic for a longer period of time^12^.

Conversely, a separate study failed to corroborate these findings. Risso et al. assessed COVID-19 infection and severity among n = 54 individuals from Italy. COVID-19 infection was ascertained by a positive COVID-19 PCR test result and all participants were assessed for PROP phenotype and PAV and AVI *TAS2R38* haplotypes. Thirty-five individuals were COVID-19 infected cases while the remaining 19 were close contacts who did not contract the disease (verified by a negative serologic test). PROP “taster” vs. “non-taster” phenotype was assessed using cotton swabs dipped in PROP solution and was shown to be strongly associated with the *TAS2R38* haplotypes. Neither PROP phenotype nor *TAS2R38* haplotype was associated with the presence or the severity of COVID-19 infection^13^. Methodological differences in the assessments of COVID-19 outcomes and phenotype/genotypes, and/or sociodemographic characteristics of the participants (as well as substantially different sample sizes) likely contribute to the different observations between these two prior studies. It is important to highlight that Barham et al. assessed COVID-19 outcomes according to phenotype as opposed to genotype.

Given the lack of consistent evidence linking *TAS2R38* haplotypes (or T2R38 phenotypes) and COVID-19 infection/severity, previous reports of differences in COVID-19 outcomes according to *TAS2R38* haplotypes (or its phenotypic expression) should be interpreted with caution, but it is important to consider that a role of *TAS2R38* in innate immunity is biologically plausible. Bitter taste receptors have been identified in various extra-oral tissues and distinct cell types such as airway smooth muscle cells, airway epithelial cells and immune cells indicate its physiological functions other than chemosensory perception in the oral cavity^8,20^. Indeed, the expression of *TAS2R38* in lung tissue has been associated with chronic rhinosinusitis^21^. Moreover, the AVI homozygous diplotype has been associated with an increased risk of colorectal cancer^22^ while the PAV homozygous diplotype has been associated with longevity^23^. These patterns could be due in part to links between *TAS2R38* haplotypes and food preferences, as well as immunity-related factors such as bacteria detection, antimicrobial actions, and ability to eliminate xenobiotics in the gut^21,22,24^. The present study (using the CLSA COVID-19 Questionnaire Sample) observed an association between *TAS2R38* haplotype and asthma, which was most frequently reported by [P]AV homozygotes and remained significant upon correction for multiple testing. This observation is notable since a previous study reported upregulation of T2Rs (bitter taste receptors) among children with severe asthma^25^. Therefore, further investigation is warranted to better understand the physiological relevance of T2R38 in extra-oral tissues and in human innate immunity.

Other relevant considerations pertinent to the present investigation should be considered. CLSA participants were community-dwelling individuals, while the participants in Barham et al.’s study were clinic/tertiary hospital patients as well as health care workers who had occupational exposure to COVID-19 (average age ~ 45 years old). In addition, the CLSA samples were comprised of individuals of rather high socioeconomic status who were at higher risk of more severe COVID-19 complications due to older age (~ 69 years old on average). Given differences in the sample characteristics, self-isolation practices are important to consider, which were likely stronger among the CLSA participants. Self-isolation could also explain the low level of infection rate observed in the CLSA COVID-19 Questionnaire and Seroprevalence samples (0.5% and 2.0%, respectively). While these percentages are somewhat in line with the estimated Canadian average prevalence of 1.1% around the time of data collection^26^, the true prevalence of infection may have been underestimated due to issues with COVID-19 testing^27^. Younger individuals, healthcare workers, visible minorities, and individuals living in a multi-unit dwelling are characteristics that have been identified as having a higher likelihood of COVID-19 infection in Canada^28^. Research on Canadian experiences during the pandemic indicate that much of the population adhered well to public health directives particularly during the early pandemic waves^29^, which appeared to be more challenging in other geographical locations^30^. Nevertheless, subgroups with lower socioeconomic status may not have been able to practice self-isolation to a similar extent due to considerations with income and employment, but these subgroups appear to be under-represented in CLSA. Meanwhile, the excluded respondents in both CLSA samples were at higher risks of having COVID-19 comorbidities, and probably were less likely to participate in the CLSA COVID-19 studies.

In addition to evidence surrounding self-isolation practices in Canada, data from the Government of Canada are also useful to critically examine the present findings obtained from the CLSA COVID-19 Seroprevalence Study sample. Mass vaccination against SARS-CoV-2 began on December 14, 2020 with older-aged adults (65 years and up) and other subgroups at high risk of severe complications due to COVID-19 infection^31^. Canada has one of the world’s highest rates of vaccination uptake for the primary series and boosters^32^. Since the CLSA COVID-19 Seroprevalence Study collected data between November 2020 and July 2021, there is an inability to differentiate between prior SARS-CoV-2 infection and vaccination in much of the Seroprevalence Sample. The availability of the CLSA COVID-19 Questionnaire Study is advantageous given that the final questionnaire data captured information mostly before the start of the mass vaccination efforts. However, the self-reported nature of the data along with the consideration of self-isolation practices must be considered in the interpretation of the present results.

Strengths of the present study include use of data from a national cohort, the evaluation of COVID-19 infection status using serology results for one sample, and data on several features of COVID-19 symptomatology. Nevertheless, the findings should be interpreted with caution due to several limitations. As discussed above, the limited number of COVID-19 cases compared with national statistics at the same time suggests either inaccuracy in data or uniqueness of the study participants compared to the population at large. Indeed, individuals who did not participate in the CLSA COVID-19 sub-studies differed in several characteristics from those who participated, suggesting presence of selection or attrition bias in the present samples. Moreover, due to the limited sample of cases, it is possible that this investigation was underpowered to detect significant associations between the *TAS2R38* haplotypes of interest and COVID-19 outcomes. The symptomatology data were self-reported (along with COVID-19 infection status in the CLSA COVID-19 Questionnaire sample). Lastly, the available CLSA samples were analyzed separately because the structure and wording of questions related to COVID-19 symptomatology were not identical, which prohibited merging of the two samples to perform one common analysis. As a result, a portion of the two study samples was comprised of the same individuals who participated in both sub-studies.

Findings from the present study suggest that common *TAS2R38* haplotypes are not related to COVID-19 susceptibility or symptoms, but additional research is warranted to determine whether the haplotypes or related phenotypes are relevant in viral infection symptomatology. While the gene is well known for playing a role in bitter taste sensitivity, prior evidence surrounding *TAS2R38*’s links with disease susceptibility and longevity, along with knowledge of the gene’s function and tissue expression, support the biological plausibility for a relationship with innate immunity. Future studies may benefit from incorporating systems biology approaches, including genomics, proteomics, metabolomics, and the microbiome, to improve the scientific understanding of the role of *TAS2R38* and/or related phenotypes in innate immunity and related biological pathways.

## Materials and methods

### Study design and population

The CLSA is a large, national, long-term study with 51,338 respondents between the ages of 45 and 85 years at baseline who will be followed longitudinally. Baseline data were collected between 2011 and 2015. A large subset referred to as the Comprehensive Cohort (n = 30,097) provided detailed sociodemographic, lifestyle, and medical data, as well as genome-wide genotyping^33^. The details of the CLSA are provided elsewhere^34^. In rapid response to the pandemic caused by the severe acute respiratory syndrome coronavirus 2 (SARS-CoV-2), the CLSA initiated three COVID-19 studies between 2020 and 2021 to explore the impacts of the pandemic on older adults (COVID-19 Questionnaire Study, COVID-19 Seroprevalence (Antibody) Study, and COVID-19 Brain Health Study)^35^. The current investigation utilized data from both the COVID-19 Questionnaire Study (comprised of self-reported data collected by questionnaires) and COVID-19 Seroprevalence (Antibody) Study (comprised of self-reported data collected by a separate questionnaire and serology from a blood sample). Genetic data from the CLSA Comprehensive Cohort were merged with these COVID-19 data sources to evaluate *TAS2R38* diplotypes in analyses. Two common single nucleotide polymorphisms (SNPs) from *TAS2R38* were available from the genome-wide assays: rs1726866 (V262A) and rs10246939 (I296V). Participants were categorized into three *TAS2R38* diplotypes based on these SNPs: [P]AV/[P]AV (subsequently referred to as “[P]AV homozygotes”), [P]AV/[A]VI (subsequently referred to as “heterozygotes”), and [A]VI/[A]VI (subsequently referred to as “[A]VI homozygotes”). The bracketed letter indicates the inferred variant that was not available from the genetic data, and thus in the present study, [P]AV is analogous to the PAV haplotype and [A]VI is analogous to the AVI haplotype.

### CLSA COVID-19 questionnaire study and seroprevalence study

A series of questionnaires were collected as part of the COVID-19 Questionnaire Study over a 9-month period (April 15, 2020 to December 29, 2020) with frequencies varying from weekly, biweekly, and monthly. Questionnaires were completed online or via telephone interview. The present investigation utilized data from the final COVID-19 questionnaire (Exit Questionnaire, n = 24,114), collected between September 29, 2020 to December 29, 2020. The Exit Questionnaire took approximately 30 min to complete and captured self-reported information on COVID-19 outcomes that occurred since the start of the pandemic (March 1, 2020), including participant infection status and symptoms, healthcare use, health behaviours, and psychosocial and economic impacts of the pandemic. In addition to the Exit Questionnaire, n = 19,334 CLSA participants participated in the CLSA COVID-19 Seroprevalence (Antibody) Study. These participants completed a separate questionnaire between November 2020 to July 2021 (internet-based or telephone interview) that collected self-reported information on COVID-19 infection status and symptoms, healthcare use, health behaviours, psychosocial and economic impacts of the pandemic since it began on March 1, 2020. In addition, these participants provided a blood sample either at a CLSA data collection site or using a self-collection kit at home. Blood samples were used to test for the presence of antibodies against SARS-CoV-2. Informed consent was obtained from all CLSA participants at baseline and prior to data collection for the COVID sub-studies. All methods conducted in this investigation were carried out in accordance with the principles outlined in the Declaration of Helsinki.

For the present investigation, data from the CLSA COVID-19 Questionnaire Study and the CLSA COVID-19 Seroprevalence (Antibody) Study were analyzed separately given that the samples were not identical, but also contained substantial overlap. Upon merging with genetic data for *TAS2R38* diplotypes, a total of n = 13,825 respondents from the CLSA COVID-19 Questionnaire Study and n = 8791 respondents from the CLSA COVID-19 Seroprevalence (Antibody) Study were included in the current analyses (Supplementary Fig. S1 and S2). Ethics approval was obtained from the McGill University Faculty of Agricultural and Environmental Sciences Research Ethics Board.

### Outcome measures

#### CLSA COVID-19 questionnaire study

Participants were considered to be cases of COVID-19 infection based on their responses to two questions in the self-reported Exit Questionnaire: “Have you ever had a positive test result?” and “Have you ever been told by a health care provider that you have COVID-19, but you did NOT have a test to confirm this?” Of the 13,825 participants with a response available, n = 42 reported a positive test result for COVID-19 and an additional n = 34 reported being told they had COVID-19 by a health care provider, providing a total of n = 76 respondents considered as COVID-19 cases for analysis.

The experiences of COVID-19 symptomatology were determined based on 12 questions in the Exit Questionnaire (detailed wording of the questions are presented in Supplementary Table S1). Common symptoms included fever, dry cough (no phlegm or mucus), wet cough (with phlegm or mucus), shortness of breath or difficulty breathing, decreased sense of smell, fatigue, sore/scratchy throat, muscle and/or joint aches/pains, headache, runny or stuffy nose, sinus pain and feeling generally unwell. For each symptom, participants self-reported the degree to which they experienced it: no symptom, mild, moderate, or severe. Due to the limited sample size of cases available, mild, moderate and severe responses were combined, and a binary outcome was analyzed for each symptom representing yes/no presence of the symptom.

#### CLSA COVID-19 seroprevalence (Antibody) study

From this study, participants with COVID-19 infection were determined based on the results of the serology variable: COVID-19 antibody result interpretation. Four groups were available based on antibody result: (1) results suggest prior SARS-CoV-2 infection (n = 177); (2) results suggest prior SARS-CoV-2 infection and/or vaccination (n = 4065); (3) results suggest prior SARS-CoV-2 infection OR prior SARS-CoV-2 infection and vaccination (n = 259); (4) no antibodies to SARS-CoV-2 detected (n = 4290). Only the first group was able to be considered as COVID-19 cases, as their results did not include the uncertainty in infection status due to vaccination.

The experiences of COVID-19 symptomatology were determined based on seven questions in the seroprevalence study questionnaire (detailed wording of the questions are presented in Supplementary Table S1). Common symptoms included fever, cough, shortness of breath, decreased sense of smell, sore throat, sore muscles and headache. The questionnaire asked only about the presence of the symptoms without severity level, so a binary outcome was analyzed for each symptom representing yes/no presence of the symptom.

### Statistical analyses

Statistical analyses were conducted using SAS software (version 9.4, SAS Institute, Cary, North Caroline, USA). Sample means and frequencies for characteristics of the CLSA respondents according to *TAS2R38* diplotypes were evaluated with analysis of variance and Pearson Chi-square tests to compare continuous and categorical characteristics, respectively. Characteristics of CLSA participants with data available vs. unavailable for the present investigation were also compared using the same statistical approach to assess the potential for attrition bias (Supplementary Table S2). Some variables assessed at CLSA’s baseline assessment were not reassessed in the COVID-19 Questionnaire Study (body mass index, self-reported ethnicity and highest level of education). In these cases, baseline data were used in these comparisons. Pearson Chi-square tests (or Fisher’s Exact test where appropriate) were used to assess differences in COVID-19 infection status, comorbidities, and symptomatology according to *TAS2R38* diplotypes, with assessment of pairwise comparisons when overall exploratory (uncorrected) significant differences were observed. Furthermore, multivariable logistic regression analyses were used to assess associations between *TAS2R38* diplotypes and COVID-19 infection status, as well as associations between *TAS2R38* diplotypes and experiences of common COVID-19 symptomatology. The regression models were adjusted for age, sex, and the first five principal components of ancestry to account for population stratification. *p*-values are two-sided and due to multiple testing statistical significance was set at FDR corrected *P* < 0.05^36^. However, given the exploratory nature of this investigation, both uncorrected (i.e. exploratory) and FDR corrected *p*-values are reported.

### Supplementary Information

Supplementary Information.

## Data Availability

Data are available from the Canadian Longitudinal Study on Aging (www.clsa-elcv.ca) for researchers who meet the criteria for access to de-identified CLSA data.
