# Supplementary material for: TAS2R38 haplotypes, COVID-19 infection, and symptomatology: a cross-sectional analysis of data from the Canadian Longitudinal Study on Aging
Source: Sci Rep. 2024 Feb 26;14:4673. doi: 10.1038/s41598-024-55428-4 (PMC10897136; doi:10.1038/s41598-024-55428-4)
Supplement: Supplementary file 1 — Supplementary Information. [file 41598_2024_55428_MOESM1_ESM.docx]

Meng et al. *TAS2R38* haplotypes, COVID-19 infection, and symptomatology: A cross-sectional analysis of data from the Canadian Longitudinal Study on Aging

**Supplementary Material**

**Contents**

**Supplementary Figure S1: Flowchart of CLSA COVID-19 Questionnaire Study respondents with data available for analyses.**

**Supplementary Figure S2: Flowchart of CLSA COVID-19 Seroprevalence (Antibody) Study respondents with data available for analyses.**

**Supplementary Table S1: Detailed wording of questions in the CLSA COVID-19 Exit Questionnaire and CLSA COVID-19 Seroprevalence (Antibody) Study Questionnaire.** For more information about the CLSA COVID-19 Exit Questionnaire, see CLSA data support material: <https://www.clsa-elcv.ca/doc/4271>. For more information about CLSA COVID-19 Seroprevalence Study Questionnaire, see CLSA data support material: <https://www.clsa-elcv.ca/doc/4362>.

**Supplementary Table S2: Characteristics of included and excluded CLSA respondents in the CLSA COVID-19 Exit Questionnaire and CLSA COVID-19 Seroprevalence (Antibody) Study.** Values presented are n (%) except for age and body mass index (BMI), which are presented as mean ± standard deviation. Responses to sex, age and current smoking status used in the COVID-19 Questionnaire Study were assessed in the CLSA COVID-19 Baseline Questionnaire. Self-reported ethnicity, highest level of education, province of recruitment and BMI used in in the COVID-19 Questionnaire Study were assessed in the CLSA baseline Comprehensive Cohort assessment. Responses to province of recruitment in the COVID-19 Seroprevalence Study were assessed in the CLSA baseline Comprehensive cohort assessment. Immune suppressed in the COVID-19 Questionnaire Study included having autoimmune disease/HIV, received an organ, bone marrow or stem cell transplant.

**Supplementary Figure S1: Flowchart of CLSA COVID-19 Questionnaire Study respondents with data available for analyses**

CLSA COVID-19 Questionnaire Study

n=28,565

Did not complete CLSA COVID-19 Exit Questionnaire

n=4,451

Respondents with responses for COVID-19 Exit Questionnaire

n=24,114

Did not participate in the CLSA Comprehensive Cohort data collection

n=8,570

Respondents with responses for CLSA baseline Comprehensive Cohort data

n=15,544

Excluded due to genetic data considerations:

Missing genotyping data: n=1,589

Missing rs1726866: n=44

Missing rs10246939: n=32

Rare haplotypes: n=54

Respondents available for the COVID-19 Exit Questionnaire analysis

n=13,825

**Supplementary Figure S2: Flowchart of CLSA COVID-19 Seroprevalence Study respondents with data available for analyses**

CLSA COVID-19 Seroprevalence Study

n=19,334

Excluded due to genetic data considerations:

Missing genotyping data: n=9,704

Missing rs1726866: n=33

Missing rs10246939: n=21

Rare haplotypes: n=36

Respondents with responses for analysis

n=9,540

Excluded due to missing SARS-CoV-2 infection-induced seroprevalence data

n=749

Respondents with SARS-CoV-2 infection-induced seroprevalence data

n=8,791

**Supplementary Table S1: Detailed wording of questions in the CLSA COVID-19 Exit Questionnaire and CLSA COVID-19 Seroprevalence (Antibody) Study Questionnaire**

| CLSA COVID-19 Exit Questionnaire | Have you experienced any of the following symptoms since March 1, 2020 and, if yes, how would you rate these symptoms?  (Selected relevant symptoms: fever, dry cough (no phlegm or mucus), wet cough (with phlegm or mucus), shortness of breath or difficulty breathing, decreased sense of smell, fatigue, sore/scratchy throat, muscle and/or joint aches/pains, headache, runny or stuffy nose, sinus pain and feeling generally unwell)  Each symptom is self-reported with the degree to which they experienced: no, mild, moderate and severe except for fever (yes/no). |
| --- | --- |
| CLSA COVID-19 Seroprevalence Study Questionnaire | Did you have any of the following symptoms between January 2020 and present?  (Selected relevant symptoms: fever, cough, shortness of breath, decreased sense of smell, sore throat, sore muscles and headache)  Each symptom is self-reported with the experience of the symptom or not. |

**Supplementary Table S2: Characteristics of included and excluded CLSA respondents in the CLSA COVID-19 Questionnaire Study and CLSA COVID-19 Seroprevalence (Antibody) Study**

|  | **COVID-19 Questionnaire Study** | | | **COVID-19 Seroprevalence Study** | | |
| --- | --- | --- | --- | --- | --- | --- |
|  | **Included (n=13,825)** | **Excluded (n=14,740)** | **p-value** | **Included (n=8,791)** | **Excluded (n=10,543)** | **p-value** |
| **Sex** |  |  | 0.03 |  |  | < 0.0001 |
| Males | 6666 (48.2%) | 6913 (46.9%) |  | 4377 (49.8%) | 4915 (46.5%) |  |
| Females | 7159 (51.8%) | 7827 (53.1%) |  | 4414 (50.2%) | 5628 (53.4%) |  |
| **Age** | 68.9 ± 9.4 | 68.7 ± 9.9 | 0.07 | 69.1 ± 9.3 | 69.0 ± 9.6 | 0.75 |
| **BMI (kg/m^2^)** | 27.9 ± 5.2 | 28.2 ± 5.7 | 0.0011 | 26.8 ± 1.2 | 27.4 ± 1.2 | < 0.0001 |
| Missing (n) | 24 | 10050 |  | 319 | 544 |  |
| **Self-reported Ethnicity** |  |  | < 0.0001 |  |  | < 0.0001 |
| Caucasians | 13434 (97.2%) | 4455 (94.6%) |  | 8399 (96.4%) | 9875 (95.5%) |  |
| Non-Caucasians | 391 (2.8%) | 253 (5.4%) |  | 344 (3.9%) | 588 (5.6%) |  |
| Missing (n) | 0 | 10032 |  | 48 | 80 |  |
| **Current Smoking status** |  |  | 0.0012 |  |  | < 0.0001 |
| Current smoker | 810 (5.9%) | 980 (6.9%) |  | 391 (4.5%) | 683 (6.6%) |  |
| Non-smoker | 12865 (94.1%) | 13275 (93.1%) |  | 8306 (95.5%) | 9629 (93.4%) |  |
| Don’t know/No answer (n) | 28 | 26 |  | 1 | 0 |  |
| Prefer not to answer (n) | 8 | 7 |  | 4 | 8 |  |
| Missing (n) | 114 | 452 |  | 89 | 223 |  |
| **Highest level of education** |  |  | 0.88 |  |  | < 0.0001 |
| Less than secondary school graduation | 560 (4.1%) | 196 (4.2%) |  | 345 (4.0%) | 660 (6.4%) |  |
| Secondary school graduation, no post-secondary graduation | 2150 (15.6%) | 741 (15.8%) |  | 1280 (14.7%) | 1910 (18.5%) |  |
| Post-secondary degree/diploma | 11098 (80.3%) | 3762 (80.0%) |  | 7051 (81.3%) | 7739 (75.1%) |  |
| Don’t know/No answer (n) | 0 | 0 |  | 18 | 19 |  |
| Prefer not to answer (n) | 0 | 0 |  | 15 | 14 |  |
| Missing (n) | 17 | 10041 |  | 82 | 201 |  |
| **Province of recruitment** |  |  | < 0.0001 |  |  | < 0.0001 |
| Atlantic | 2335 (16.9%) | 805 (17.1%) |  | 1631 (18.6%) | 350 (19.1%) |  |
| British Columbia | 2885 (20.9%) | 1017 (21.6%) |  | 1786 (20.3%) | 367 (20.0%) |  |
| Ontario | 3204 (23.2%) | 956 (20.3%) |  | 1979 (22.5%) | 304 (16.6%) |  |
| Prairie | 2847 (20.6%) | 880 (18.7%) |  | 1706 (19.4%) | 290 (15.8%) |  |
| Quebec | 2554 (18.5%) | 1050 (22.3%) |  | 1689 (19.2%) | 521 (28.5%) |  |
| Missing (n) | 0 | 10032 |  | 0 | 8711 |  |
| **Comorbidities** |  |  |  |  |  |  |
| Asthma | 1433 (10.5%) | 1365 (9.5%) | 0.007 | 841 (9.7%) | 978 (9.5%) | 0.64 |
| Don’t know/No answer (n) | 67 | 75 |  | 43 | 41 |  |
| Prefer not to answer (n) | 15 | 17 |  | 8 | 7 |  |
| Missing (n) | 84 | 315 |  | 86 | 219 |  |
| Chronic Lung Diseases | 813 (6.0%) | 900 (6.3%) | 0.25 | 309 (3.6%) | 549 (5.4%) | < 0.0001 |
| Don’t know/No answer (n) | 67 | 75 |  | 63 | 64 |  |
| Prefer not to answer (n) | 15 | 17 |  | 0 | 2 |  |
| Missing (n) | 84 | 315 |  | 86 | 219 |  |
| Diabetes | 1522 (11.1%) | 1841 (12.8%) | < 0.0001 | 970 (11.2%) | 1526 (14.8%) | < 0.0001 |
| Don’t know/No answer (n) | 67 | 75 |  | 37 | 31 |  |
| Prefer not to answer (n) | 15 | 17 |  | 1 | 1 |  |
| Missing (n) | 84 | 315 |  | 86 | 219 |  |
| Hypertension | 4725 (34.6%) | 5235 (36.5%) | 0.0007 | 2630 (30.4%) | 3144 (30.6%) | 0.78 |
| Don’t know/No answer (n) | 67 | 75 |  | 51 | 44 |  |
| Prefer not to answer (n) | 15 | 17 |  | 2 | 1 |  |
| Missing (n) | 84 | 315 |  | 86 | 219 |  |
| Heart Diseases | 1528 (11.2%) | 1679 (11.7%) | 0.17 | 634 (7.4%) | 943 (9.3%) | < 0.0001 |
| Don’t know/No answer (n) | 67 | 75 |  | 107 | 135 |  |
| Prefer not to answer (n) | 15 | 17 |  | 2 | 1 |  |
| Missing (n) | 84 | 315 |  | 86 | 219 |  |
| Cancer | 1257 (9.2%) | 1396 (9.7%) | 0.13 | 1176 (13.6%) | 1531 (14.9%) | 0.0094 |
| Don’t know/No answer (n) | 67 | 75 |  | 30 | 31 |  |
| Prefer not to answer (n) | 15 | 17 |  | 0 | 3 |  |
| Missing (n) | 84 | 315 |  | 86 | 220 |  |
| Immune suppressed | 970 (7.1%) | 1041 (7.2%) | 0.61 | 256 (3.0%) | 400 (3.9%) | 0.0005 |
| Don’t know/No answer (n) | 1 | 0 |  | 86 | 85 |  |
| Prefer not to answer (n) | 0 | 1 |  | 0 | 2 |  |
| Missing (n) | 84 | 315 |  | 86 | 220 |  |
